# Responses to Telomere Erosion in Plants

**DOI:** 10.1371/journal.pone.0086220

**Published:** 2014-01-21

**Authors:** Simon Amiard, Olivier Da Ines, Maria Eugenia Gallego, Charles I. White

**Affiliations:** Génétique, Reproduction et Développement, Unité Mixte de Recherche 6293, Centre National de la Recherche Scientifique - Clermont Université - Unité 1103, Institut National de la Santé et de la Recherche Médicale, Aubière, France; Tulane University Health Sciences Center, United States of America

## Abstract

In striking contrast to animals, plants are able to develop and reproduce in the presence of significant levels of genome damage. This is seen clearly in both the viability of plants carrying knockouts for key recombination and DNA repair genes, which are lethal in vertebrates, and in the impact of telomere dysfunction. Telomerase knockout mice show accelerated ageing and severe developmental phenotypes, with effects on both highly proliferative and on more quiescent tissues, while cell death in Arabidopsis *tert* mutants is mostly restricted to actively dividing meristematic cells. Through phenotypic and whole-transcriptome RNAseq studies, we present here an analysis of the response of Arabidopsis plants to the continued presence of telomere damage. Comparison of second-generation and seventh-generation *tert* mutant plants has permitted separation of the effects of the absence of the telomerase enzyme and the ensuing chromosome damage. In addition to identifying a large number of genes affected by telomere damage, many of which are of unknown function, the striking conclusion of this study is the clear difference observed at both cellular and transcriptome levels between the ways in which mammals and plants respond to chronic telomeric damage.

## Introduction

Telomere structure and DNA damage response (DDR) and repair networks are very highly conserved among eukaryotes. Studies of the DDR in animals are however complicated by the lethality of knockouts of many of the key genes. In striking contrast, Arabidopsis (and presumably other plants) is able to develop, grow and differentiate in presence of significant genome damage. This difference is both surprising and of real biological interest.

The genomes of the majority of studied eukaryotic organisms consist of linear chromosomes, and each chromosome thus has two ends. The proper replication and protection of these chromosome-ends poses particular problems to the cell and these have been solved by the evolution of a specialised nucleoprotein structure, the telomere. A number of telomeric proteins have been identified and these act to “cap” the telomere and to “hide” it from the cellular DNA repair and recombination machinery. Vertebrate telomeres are protected principally by Shelterin, a complex of six telomeric proteins (TRF1, TRF2, POT1, TIN2, TPP1 and RAP1). These prevent inappropriate recombination and fusion between telomeres, and also play roles in telomere replication and regulation of telomere length [1], [2]. Although its telomeric DNA is similar to that of mammals, *Saccharomyces cerevisiae* has a somewhat simpler protection complex consisting principally of the Cdc13, Stn1 and Ten1 proteins (referred to as the CST complex) [3]–[5].

In *Arabidopsis thaliana* and in plants in general, only a subset of the vertebrate shelterin components has been identified (reviewed by [6]). The implication of CST in telomere maintenance (either by direct protection or help in replication) is however clearly established [7]–[9]. Plant telomeres thus seem to be at the crossroads between *S*. *cerevisiae*, which has only CST as a capping complex, and vertebrates, which use both Shelterin and the CST complex for telomere capping and correct telomeric replication [10], [11].

Unprotected telomeres are recognised by the cell as DNA double-strand breaks (DSB) and lead to the activation of the DNA-damage response (DDR), chromosome fusions, rearranged chromosomes and cell death. In mammals, this signalling is carried out by three protein kinases belonging to the PI3K-like protein kinases (PIKK) family: ATM, ATR and DNA-PKcs. Activated PIKK phosphorylate many targets, activating pathways for the maintenance of genome integrity and the elimination of genetically unstable cells, mainly through the activation of the p53 transcription factor [12], [13]. This role is fulfilled by the SOG1 transcription factor in Arabidopsis [14]. ATM and ATR have been characterized in Arabidopsis, but no DNA-PKcs gene has been identified [15]–[17]. Studies of the roles of ATM and ATR in H2AX phosphorylation show that one or both of these are necessary and sufficient for activation of the DDR in Arabidopsis, confirming the absence of a third kinase [18]. Only ATR is required for signalling of deprotected telomeres in Arabidopsis *cst* mutants, while principally ATM, but also ATR, is activated by eroded telomeres in *tert* mutant plants [19]. ATR is required for the induction of programmed cell death allowing the maintenance of genomic integrity through elimination of genetically unstable cells [19], [20].

The specialised telomere structure also acts to counteract DNA erosion arising from the inability of DNA polymerases to fully replicate the ends of linear chromosomes. This is compensated for by the telomerase, a specialised reverse transcriptase that extends chromosome 3′ DNA ends by adding repeats of telomeric DNA using its RNA subunit as template. In the absence of telomerase, telomere erosion acts as a biological “clock”, limiting the proliferative potential of cells and playing a major role in cellular ageing and protection against cancer [21]. Absence of the telomerase reverse transcriptase (TERT) in Arabidopsis leads to the progressive erosion of telomeric DNA sequences, which, in turn, results in telomere uncapping and increasingly severe genetic instability accompanied by visible developmental defects and reduced fertility in the fourth or fifth mutant generations. These become progressively more severe in succeeding generations, resulting in problems in growth and development and in complete sterility by the tenth or eleventh generation [22]. The effects of telomere erosion in mammals are also dramatic. Mice deficient for TERT exhibit reduced fertility and progressive defects in highly proliferative organs in the 3^rd^ generation and embryonic developmental defects and sterility in the 6^th^ generation [23]–[26]. The most striking difference is that plants harbouring short telomeres have an extended life span and remain metabolically active while telomere dysfunction in mice induces metabolic and mitochondrial compromise [27].

To date, the specific plant mechanisms involved in this response are not known. Taking advantage of the progressive appearance of the phenotypic effects in succeeding generations of Arabidopsis *tert* mutants, we present here phenotypic and whole-transcriptome RNAseq analyses separating the effects of the absence of telomerase (in both early- and late-generation *tert* mutants) and the resulting genome damage (only in late-generations). Our data provide a strikingly different picture from that reported in the study of telomerase mutant mice [27].

## Materials and Methods

### Plant Material and Growth Conditions

The T-DNA insertion Arabidopsis telomerase (*tert*) mutant and PCR-based genotyping have been described previously (Fitzgerald et al., 1999). All plants come from an original heterozygous *tert* mutant plant.

Plants were grown under standard conditions: seeds were stratified in water at 4°C for 2 days and grown *in vitro* on 0.8% agar plates, 1% sucrose and half-strength MS salts (M0255; Duchefa Biochemie, http://www.duchefa-biochemie.nl), with a 16-h light/8-h dark cycle, at 23°C with 45–60% relative humidity.

### DAPI Staining of Mitosis

Seven days after germination, root tips were fixed for 45 min in 4% paraformaldehyde in PME (50 mM PIPES, pH 6.9, 5 mM MgSO4, and 1 mM EGTA) and then washed 3 times for 5 minutes each in PME. Root tips were then digested for 30 min in 1% (w/v) cellulase, 0.5% (w/v) cytohelicase, and 1% (w/v) pectolyase (from Sigma-Aldrich; Refs. C1794, C8274, and P5936) solution prepared in PME and then washed 3 times 5 minutes in PME. Digested root tips were gently squashed onto slides (Liu et al., 1993), air dried, and mounted using Vectashield mounting medium with 1.5 µg/mL DAPI (Vector Laboratories) and observed by fluorescence microscopy. Images were further processed and enhanced using Adobe Photoshop software.

### Cell Death Assay

Seven days after germination, seedlings were immersed in Propidium Iodide solution (5 µg/ml in water) for 1 min and rinsed three times with water. Root tips were then transferred to slides in a drop of water and covered with a cover slip for observation under the fluorescence microscope with a Zeiss filter set 43HE (adapted from Curtis and Hays, 2007).

### Flow Cytometry Analysis

Nuclei were prepared with the Cystain UV Precise P kit (#05-5002; Partec GmbH, Germany. http://www.partec.com), following the manufacturer's instructions. Briefly, nuclei of approximately 20 seven-day-old seedlings were chopped with a razor blade in 200 µl of Cystain UV Precise P extraction buffer, 800 µl of Cystain UV Precise P staining buffer was added and the sample filtered through 30 µm nylon mesh. Flow cytometry was performed using an Attune Acoustic Focusing Cytometer (Life Technologies), following the manufacturer’s protocols. Results were analysed using the Attune Cytometric Software version 1.2.5.

### Determination of the Mitotic Index

Roots were fixed in a solution of 4% paraformaldehyde in PBS for 45 min, washed twice in PBS/1% (v/v) Tween-20, stained for 30 min in Hoechst 33258 (3 µg/ml), rinsed in PBS/Tween, and mounted under cover slips in 40% glycerol. The roots were analysed for mitotic stages (metaphase and anaphase/telophase) using fluorescence microscopy with Zeiss filter set #49.

### EdU Pulse-chase

Arabidopsis seedlings were germinated as usual and after 7 days were transferred to liquid medium containing 10 µM of EdU for 2 hours. Seedlings were then rinsed twice, transferred to fresh medium containing 50 µM of thymidine (no EdU) for 0, 6, 12 or 24h and fixed in 3.7% formaldehyde. After permeabilization in 0.5% Triton X-100, EdU detection was performed with the Invitrogen Click-iT EdU Alexa Fluor 594 Imaging kit as previously described (Amiard et al., 2010). Root tips were fixed for 45 min in 4% paraformaldehyde in a solution of 1 X PME (50 mM Pipes, pH 6.9, 5 mM MgSO4, 1 mM EGTA) and then washed three times for 5 min in 1X PME. Tips were digested for 1 h in a 1% (w/v) cellulase, 0.5% (w/v) cytohelicase, 1% (w/v) pectolyase (Sigma-Aldrich; Refs. C1794, C8274, P5936) solutions prepared in PME and then washed three ×5 min in PME. They were then gently squashed onto slides as described previously (Liu et al., 1993), air dried, and stored at −80°C.

### RNA Extraction

RNA was extracted from seven day-old plantlets with TriZol reagent (Invitrogen) and purified with the RNeasy plant mini kit (Qiagen) as recommended by the manufacturers.

### Quantitative RT-PCR

Total RNA was prepared using RNeasy kit (QIAGEN) as suggested by the manufacturer and 2 µg reverse transcribed with MMLV reverse transcriptase (Promega). Q-PCR was carried out using primers: 5′-TGCATCCATTAAGTTGCCCTGTG-3′ and 5′-TAGGCTGAGAGTGCAGTGGTTC-3′ for *BRCA1* (At4G21070), 5′-ATGCTACTCTGGCACGGTTCAC-3′ and 5′-AGGAGGAGCTATTCGCAGACCTTG-3′ for *PARP1* (At4G02390), and 5′-CGAGGAAGGATCTCTTGCAG-3′ and 5′- GCACTAGTGAACCCCAGAGG-3′ for *RAD51* (At5G20850). Reactions were run on a Roche “LightCycler® 480 Real-Time PCR System” using 55°C primer annealing and 15s extension using LightCycler® 480 DNA SYBR Green I Master (Roche) according to the manufacturer’s instructions. Reactions were performed in triplicate using UBQ10 as the endogenous control. Expression levels for each genotype were averaged and compared with that of wild type.

### High-Throughput Sequencing of mRNA Using the SOLEXA Technology

RNAseq analysis was carried out by Fasteris S.A. (Plan-les-Ouates, Switzerland). Briefly, ten micrograms of total RNA per sample was used to generate the cDNA Colony Template Libraries (CTLs) for high-throughput DNA sequencing using SOLEXA technology (Fasteris Genome Analyzer Service). Poly(A) transcripts were purified, and double-stranded cDNA synthesis was performed using oligo(dT) priming for first-strand synthesis. cDNA was fragmented into 50- to 200-bp fragments through nebulization, followed by end repair, addition of 3′ adenine nucleotides, ligation of adapters, gel purification to isolate fragments of 150 to 500 bp, and PCR amplification. For quality control analysis, an aliquot of each CTL was cloned into the TOPO plasmid, and 5 to 10 clones were sequenced using capillary sequencing. The CTLs were sequenced on the Illumina Genome Analyzer, generating 18 to 20 million reads of 100 bases in length per sample. Two replicate samples from independently conducted biological experiments were run for each genotype. The standard Illumina analysis pipeline was used for collecting raw images and base calling to generate sequence files, which were used as primary data files for further analysis.

### Data Analysis

Raw sequence files from the Illumina pipeline were used for alignment against the TAIR10 *Arabidopsis* genome sequence release using BWA software. First, the original 100-mers were aligned with a tolerance of up to five mismatches. On average, we found a unique hit for 85% of the reads, giving approximately 16 million reads per library mapped uniquely to the *Arabidopsis* genome. Seqmonk software was used for visualization and analysis of mapped sequence. The genes for which less than 20 hits were recorded in all samples were discarded from the data set. Comparisons of relative levels of transcripts in wild type, *tertG2* and *tertG7* plants in two independent experiments were carried out as described in the main text. Gene ontology classification of the transcripts was done according to classical gene ontology categories using the web-based tool Classification Super-viewer (http://bar.utoronto.ca).

## Results/Discussion

### Phenotypic Analyses of Early and Late Generation *tert* Mutants

**Figure 1:**
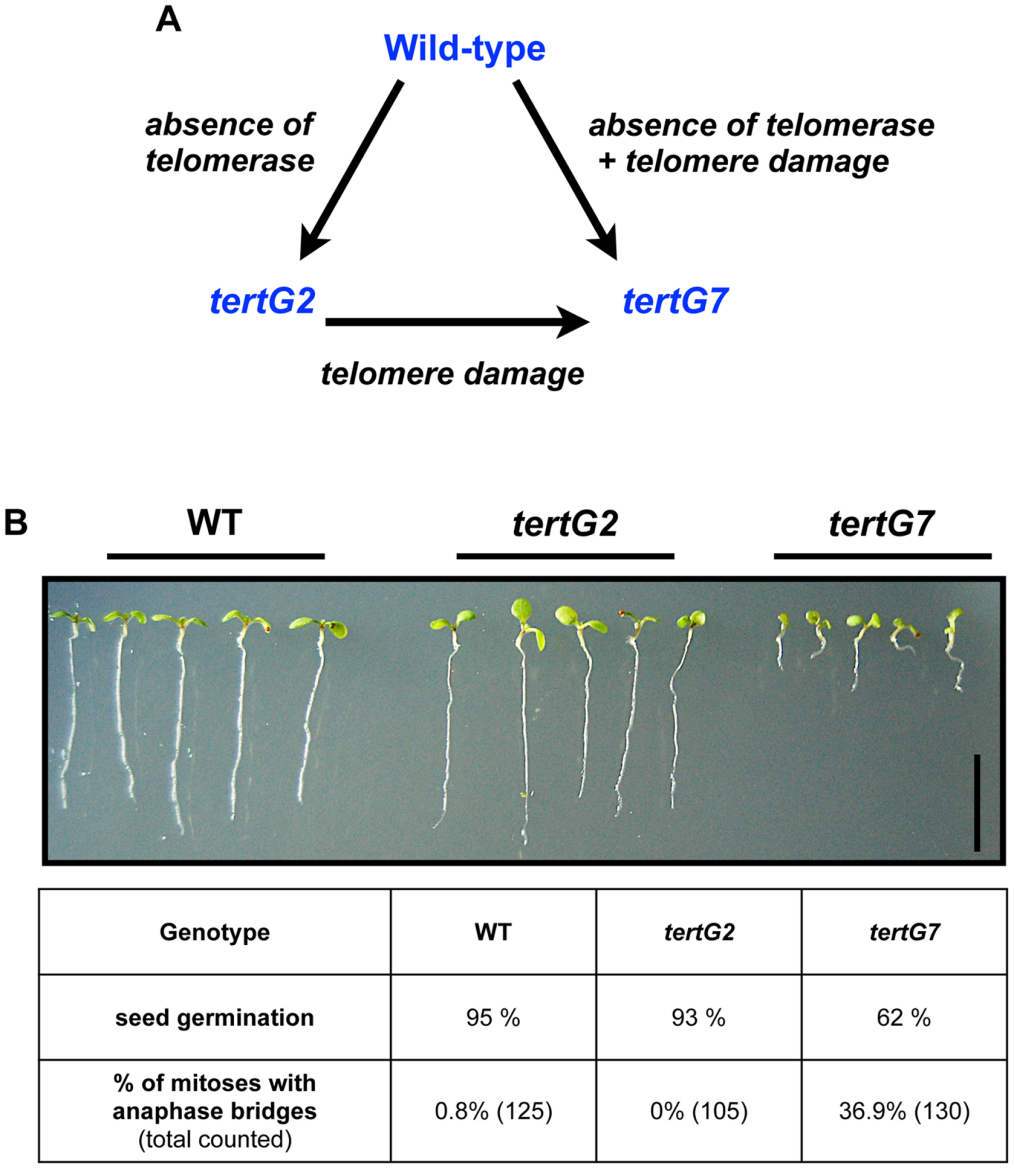
Phenotypic analysis of early and late generation of *tert* mutants. (A) Schematic description of the experimental approach. Second generation *tert* mutant plants (*tertG2*) lack telomerase but have functional telomeres, while seventh generation *tert* mutants (*tertG7*) both lack telomerase and have dysfunctional telomeres. Comparison of *tertG2*, *tertG7* and wild-type (WT) plants thus permits separation of the effects of the absence of telomerase enzyme from the consequences of telomere erosion. (B) 7-day old *tertG2* plantlets show wild-type root growth and fertility, in contrast to severely reduced root growth and poor seed germination of *tertG7* plantlets. Root meristem cells of *tertG7* plantlets also show elevated levels of mitotic anaphase chromosome bridges, in contrast to *tertG2* and wild type (WT) plantlets. Bar = 1 cm.

Early generation *tert* mutants appear phenotypically normal, while late generation *tert* plants show severe developmental defects accompanied by high levels of chromosome fusions visible as anaphase bridging in mitotic cells [22]. Comparison of Wild-Type (WT), early (*tertG2)* and late (*tertG7)* plants thus permits separation of the effects of the absence of telomerase enzyme (in *tertG2* and *tertG7*) from the consequences of the uncapped telomeres and genome damage (*tertG7* only) (Figure 1A and 1B).

Seven days after germination, *tertG2* seedlings are viable and phenotypically indistinguishable from wild type plants, while *tertG*7 seeds germinate poorly (∼ 1/3 do not germinate) and plants show severe developmental defects (Figure 1B). Cytogenetic analyses of root meristem cells confirm that these visible phenotypes are accompanied by (and presumed to result from) telomere deprotection, visible as Telomere Induced Foci (TIF) [19] and elevated levels of chromosome fusions visible as mitotic anaphase bridges (Figure 1B).

**Figure 2:**
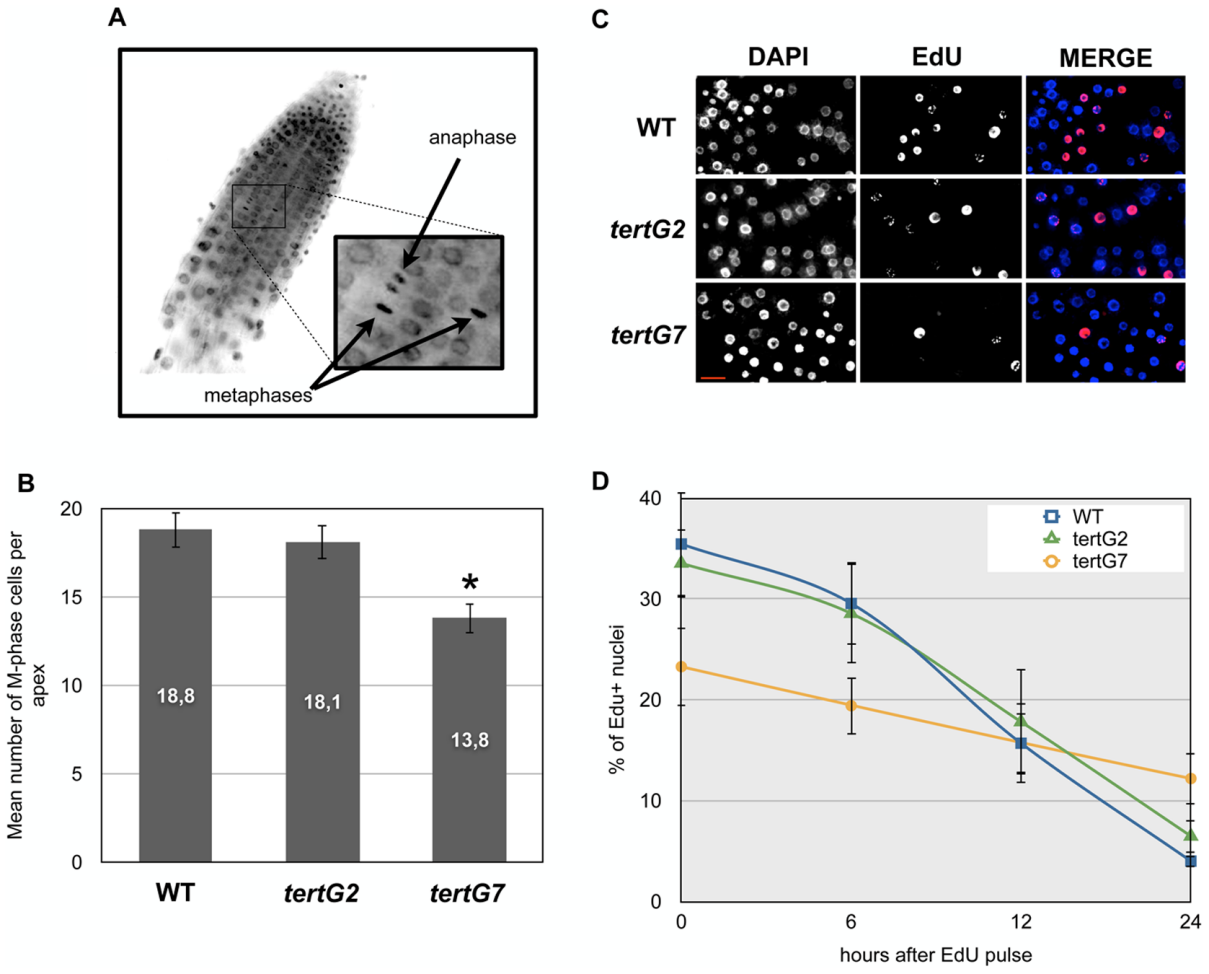
Cell Cycle Regulation in Root Tips of WT, *tertG2* and *tertG7* mutants. (A) Representative images of root tips stained with DAPI (images are representative of ten root tips) for counting M-phase (anaphase or metaphase) nuclei. (B) Mean numbers of M-phase mitotic nuclei per root tip in 7-day-old WT, *tertG2* and *tertG7* seedlings. Error bars are standard errors (n = 10) and the asterisk shows significant difference (t test; P<0.05) between the WT and *tertG7* mutants. (C) Representative images of root tip nuclei after 2h of EdU pulse. (D) The percentages of EdU+ nuclei after 0, 6, 12 or 24h are reported in the graphic (n>1000 nuclei in each condition).

As expected and in accord with the previous characterisation of late generations of *tert* mutants [22], *tertG7* plants present severe genomic instability. Notwithstanding this, the affected plants are still able to develop and we thus were able to characterise the cellular and developmental responses to telomere deprotection in *tertG2* and *tertG7* plants. Cell proliferation status was estimated through the study of mitotic index. As illustrated in Figure 2A,B, we observe a clear decrease in the numbers of mitotic figures in *tertG7* plants with respect to *tertG2* and WT plants, which do not differ significantly. To take this further, we analysed cell cycle progression through an EdU pulse/chase experiment (Figure 2C-D). EdU is a thymidine analogue that is incorporated into DNA during S-phase and EdU-subsituted DNA can be detected cytologically through a fluorescence assay. After 2h of growth in the presence of EdU, 35.4% of WT and 33.5% of *tertG2* root nuclei have detectable EdU incorporation. In *tertG7* plants, this is reduced to 23,3%. This cell cycle slow-down is confirmed by the time course of EdU dilution in subsequent divisions, which is clearly faster in WT and *tertG2* compared to *tertG7* plants. 24h after the EdU pulse, the percentage of EdU positive nuclei drops to 4% in WT and 6.5% in *tertG2*, but only to 12.2% in *tertG7*. This slowing of cell division is not surprising considering the phenotype of *tertG7* plants and is consistent with the activation of the DDR, known to provoke cell cycle arrest [18], [28], [29].

**Figure 3:**
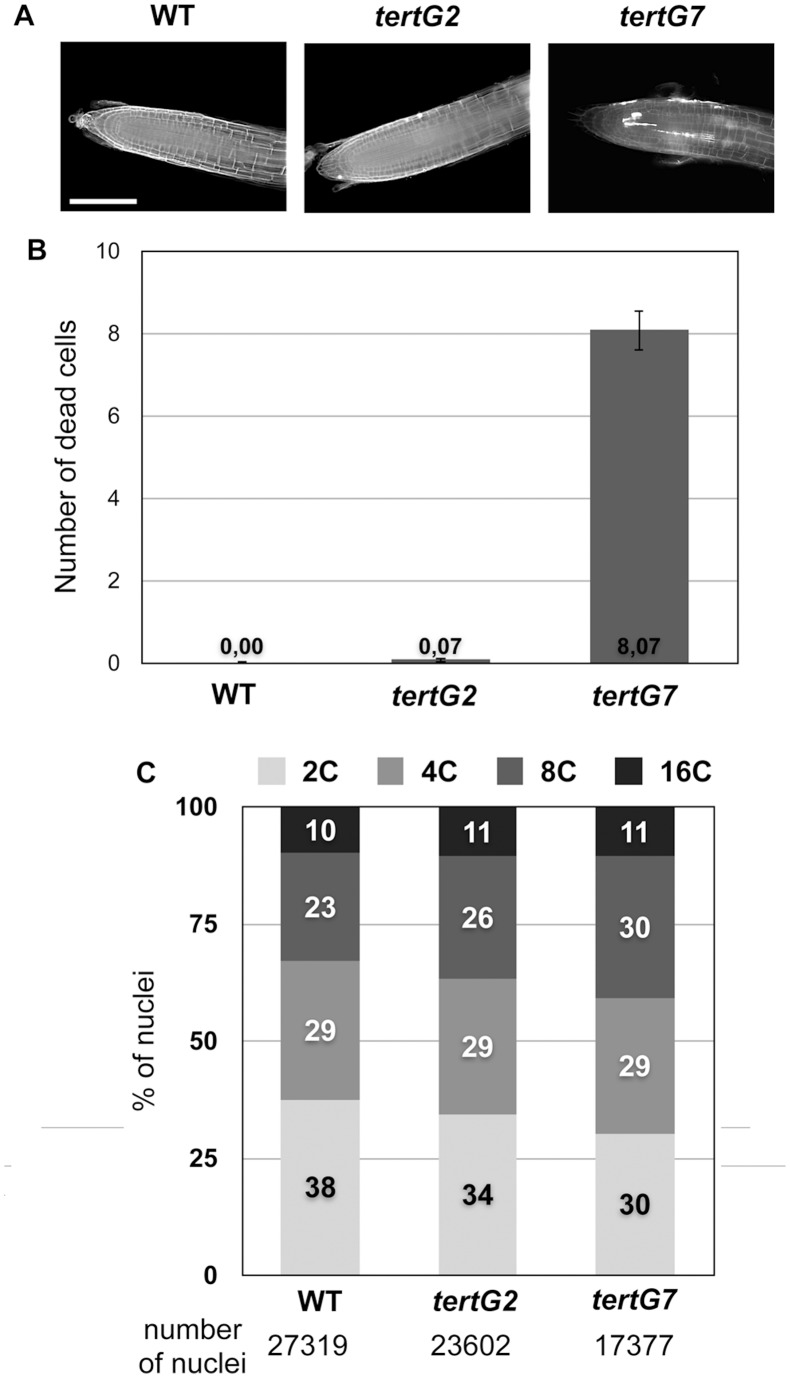
Cell death and ploidy analyses in WT, *tertG2* and *tertG7* mutants. (A) Representative images of root tips stained with Propidium Iodide (which stains dead cells). No cell death is observed in WT or in *tertG2* plants, while abundant cell death is observed in the region around the quiescent center in *tertG7* mutants. (B) Mean numbers of dead cells per root tip for 7 day-old WT, *tertG2* and *tertG7* seedlings (ten root tips for each class; error bars are standard errors). (C) Flow cytometry measurements of DNA content of DAPI stained nuclei show no significant differences in ploidy in WT, *tertG2* and *tertG7* mutant plants. The number of analysed nuclei for each class is given below the graph.

Maintenance of genomic integrity in Arabidopsis in the presence of telomere dysfunction depends upon programmed cell death in order to eliminate genetically unstable cells [19], [20]. To verify that it is also the case in *tertG7* plants, we quantified cell death by Propidium Iodide staining of root tips. As expected, we observe the appearance of high numbers of dead cells in root meristems of *tertG7* plants but not in *tertG2*, nor in WT plants (Figure 3 A,B).

Increases in ploidy are common in plant development [30] and could act to reduce the impact of chromosome instability and thus potentially explain the remarkable survival of *tertG7* plants. In support of this argument, it has recently been shown that Arabidopsis plants induce a SOG1-dependent programmed endoreduplicative response to DNA double strand breaks [31]. To test for an equivalent response to telomeric damage, we used flow cytometry to carry out ploidy analysis on nuclei of seven-day-old WT, *tertG2* and *tertG7* plantlets. The results of this analysis are presented in Figure 3C and although a small increases in ploidy are observed, the differences are not significant. This result differs from the increases in ploidy observed in plants treated with ionising radiation (IR) or DSB inducing agents [31], although it seems likely that this is more a reflection of the difference between low levels of chronic DSB (deprotected telomeres) and the high level acute damage imposed by the genotoxic treatments. In *tertG7* plants, the shortening of telomeres leading to chronic damage appears to be dealt with mainly through PCD and less through increases in ploidy.

### Global Transcriptome Analyses

**Figure 4:**
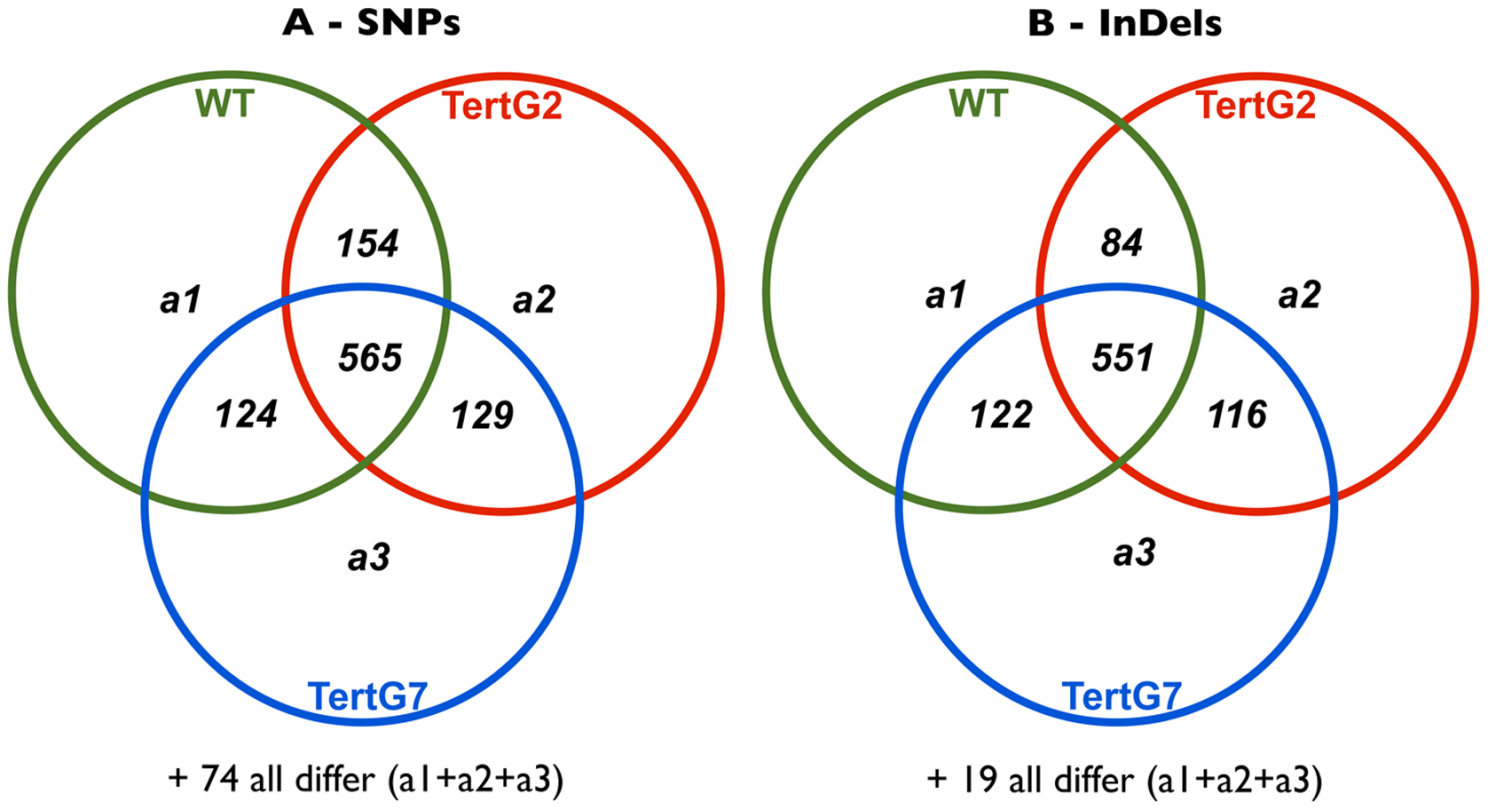
Chromosomal instability in *tertG7* plants does not induce high numbers of SNPs or InDels. Venn diagram showing the common and differential SNPs (A) or InDels (B) between WT, *tertG2* and *tertG7* from RNAseq study.

The presence of deprotected telomeres thus induces cell-cycle slow-down and programmed cell death in meristems, to permit repair of damage and to eliminate genetically unstable cells. However these mechanisms alone cannot explain the extraordinary capacity of plants to grow in presence of such damage (37% of *tertG7* root meristem mitoses show visible chromosome bridges). We thus carried out global transcriptome analyses on these plants to identify response pathways and potentially novel components of the DNA Damage Response (DDR). mRNA was isolated from wild-type, *tertG2* and *tertG7* plants and Illumina Hi-seq 2000 RNAseq analyses carried out to establish the individual and combined effects of the presence of telomere damage and the absence of the telomerase on global transcriptome patterns. The sequences were aligned to the TAIR10 reference Arabidopsis genome sequence using the BWA tool and the SEQMONK program used to identify and quantitate transcript levels of individual genes. In two independent repetitions, the RNAseq analyses yielded approximately 20 million sequences per sample, with quantifiable transcripts (at least 20 reads per transcript) from 18893 genes present in samples from both repetitions (Table S1). We note that, notwithstanding the presence of elevated levels of mitotic chromosome bridges and the continuous induction of the DDR (presence of TIFs) in *tertG7* plants, no evidence for higher levels of single nucleotide polymorphisms (SNPs) and DNA insertions/deletions (InDels) is seen in the RNAseq data from these plants (Figure 4).

**Figure 5:**
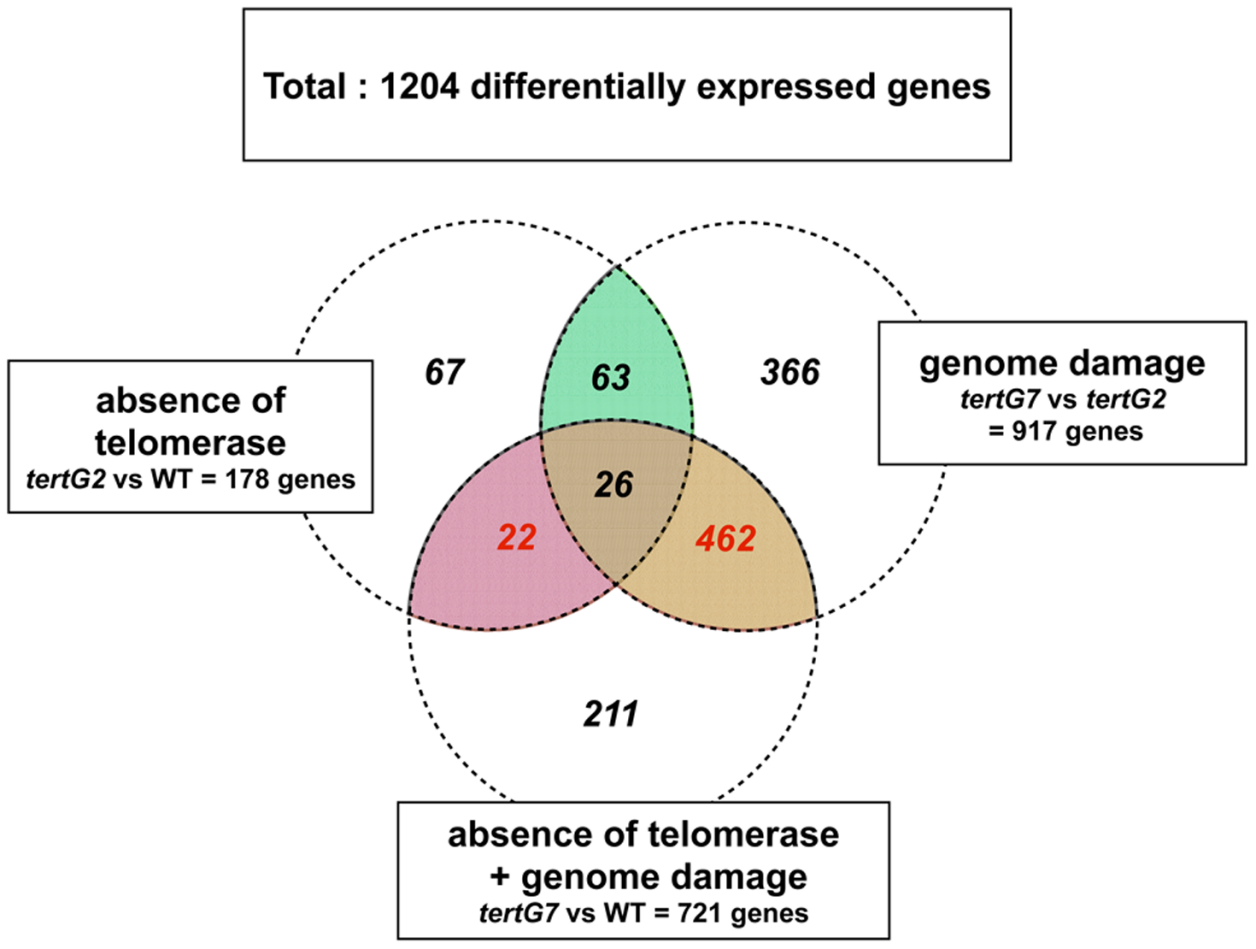
RNAseq analyses of transcriptional responses to the absence of telomerase and to telomere damage. Venn diagram presenting the results of RNAseq analyses of WT, *tertG2* and *tertG7* mutants. Numbers of genes showing differing transcription in the WT, *tertG2* and *tertG7* plants, in both of two independent experiments. The RNAseq data yielded 18893 expressed genes present in both experiments, and of these, 1204 were either up or down regulated (see text for detail).

The results of screening for genes with altered expression in the WT versus *tertG2*, WT versus *tertG7*, and/or *tertG2* versus *tertG7* plants are presented as a Venn diagram in Figure 5. For this global screen, we chose to arbitrarily select genes for which the increase (or decrease) in transcript level is at least 2-fold in one set and at least 1.5-fold in the other. In total, 1204 differentially expressed genes were identified and these were divided into the following classes (permitting genes to be present in more than one class): 178 genes for *tertG2*/WT, 917 genes for *tertG7*/*tertG2* and 721 genes for the combined effects of telomere damage and absence of telomerase (*tertG7*/WT). The corresponding lists of genes are presented in Table S2.

**Figure 6:**
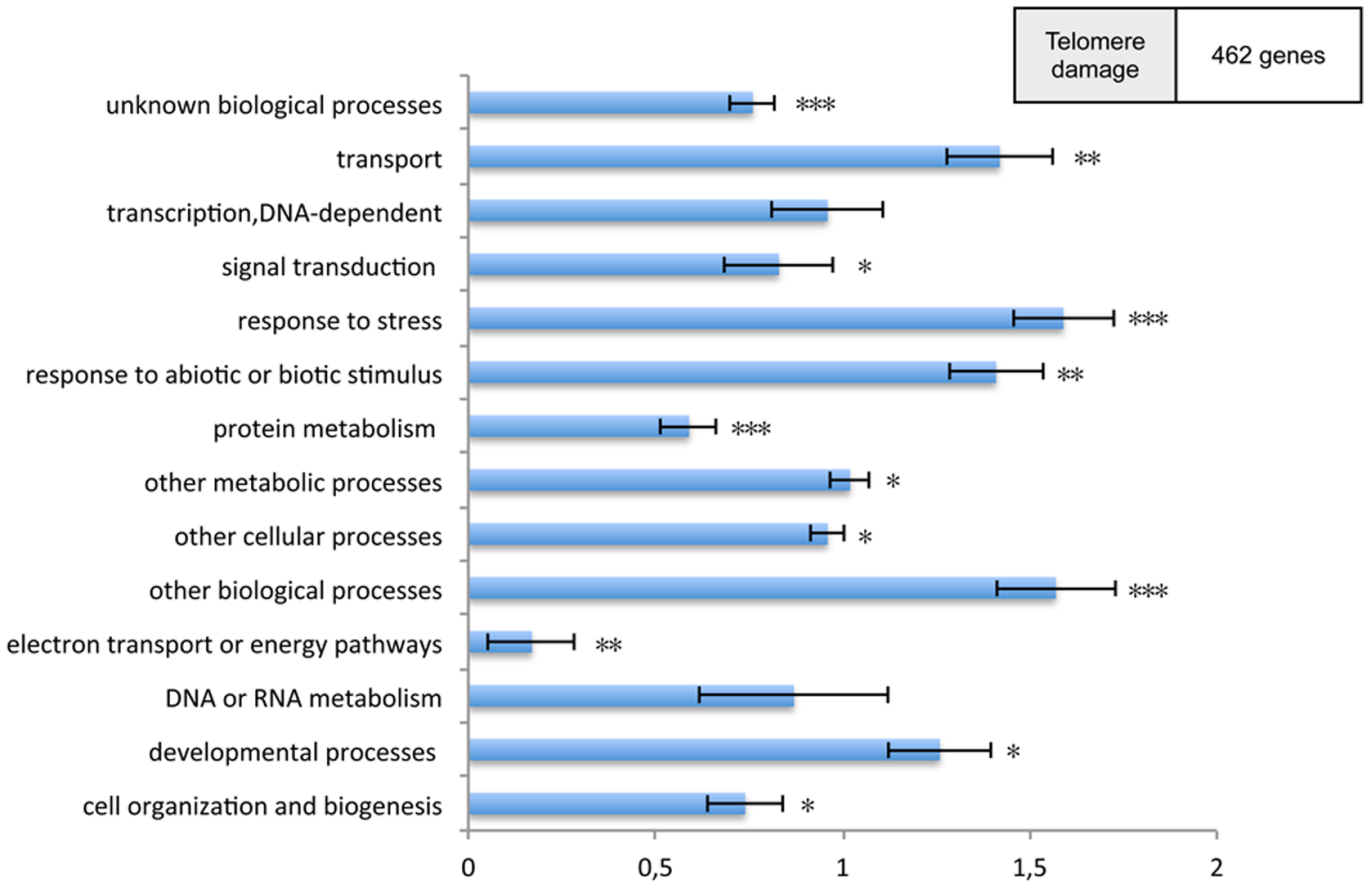
Gene ontology classification in late telomerase generation. Functional “Biological process” classification of differentially expressed transcripts in the “telomere damage” context. Gene ontology classification of the transcripts according to classical gene ontology categories using the web-based tool Classification Super-viewer (http://bar.utoronto.ca) with the “normalized class” score option. One, two and three asterisks indicate p-values below 0.05, 0.001 and 0.0001, respectively.

To further refine the selection of genes specifically affected by telomere damage (*tertG7*/WT), we excluded those affected by the absence of telomerase alone (*tertG2*/WT). This resulted in 462 genes showing altered expression specifically due to telomere damage (Figure 5 and Table S3). A similar approach yielded 22 genes showing altered expression specifically due to the absence of telomerase (Figure 5 and Table S3). The numerically significant effects on altered transcription are seen mainly in phenotypically altered *tertG7* mutant plants, in accordance with the severity of their phenotype. On the other hand, very few genes are deregulated specifically by the absence of functional telomerase. In addition to the identification of specific candidate genes of interest, we also carried out Gene Ontology (GOslim) for Biological Process (Figure 6). Although the GO classes are very general and care should be taken not to draw definite conclusions from the GO analysis, this classification can be of real utility in identifying unexpected effects. Classification of the “telomere damage responding” genes with the “normalised class” score option shows a high representation of genes encoding proteins involved in “response to stress”, “protein metabolism” and “transport”.

### Focus on Response to Stress

An universal stress response transcriptome encompassing 197 genes that are induced by a broad range of stress conditions has been established for plants [32]. Of these 197 genes, 14 are also deregulated in consequence of telomeric damage (Table S4-1), suggesting that telomere erosion triggers a specific response. As mentioned above, the Gene Ontology (GOslim) analysis revealed a significant over-representation of genes in the “response to stress” category. GOterm classification of the genes assigns 23% of “telomere damage responding” genes (106 of 462) (Table S4-2) to the “response to stress” category (compared to 16% in this category for the whole genome). Most of these genes belong to the “abiotic stresses” subclass and the “defence response” subclass was the most enriched (Table 1).

**Table 1:** GO classification of the 104 “stress” category genes deregulated in *tertG7* mutants.

GO term category	Counts
DNA or DSB repair	10
Telomere maintenance	1
**Biotic stress**	
Defence response	31
Systemic acquired and induced systemic resistance	11
Hypersensitive response	6
**Abiotic stress**	
Cellular response to starvation	18
Response to salt stress	16
Response to oxidative stress	14
Response to heat	13
Response to cold	13
Response to water deprivation	12
Response to wounding	10
Response to hydrogen peroxide	6
Response to osmotic stress	6
Response to freezing	4
Response to hypoxia	3
Response to ozone	2
SOS response	1
Cellular response to Nitric oxide	1
Response to ER stress	7

(A given gene can be classified in more than one category).

### Focus on DNA Recombination and Repair

Surprisingly, considering the ATM/ATR dependent activation of the DDR pathway in *tertG7* plants, relatively few genes related to “DNA repair and recombination” are deregulated, including the kinases ATM and ATR (Table S5). “Telomere deprotection” upregulates transcription of major homologous recombination (HR) proteins such as RAD51, PARP1 and BRCA1, in accordance with their known response to genotoxic treatments [16], [32]–[34]. The modifications in the transcriptional regulation of these three genes are confirmed by Q-RTPCR analyses (see Figure S1) and have been reported by others [20], [35], [36]. No changes were observed in transcript levels of KU80, XPF or XRCC1, involved in the non-homologous end-joining (NHEJ) or single-strand-break (SSB) DNA repair pathways [37], [38]. We also remark the downregulation of CENTRIN2, a nucleotide excision repair (NER) regulating protein, in mutants of which the NER repair defect is accompanied by enhanced levels of somatic homologous recombination (HR) [39], again supporting a preference for induction of HR. The AGO2 gene, which has recently been found to play an important role in recombination by recruiting diRNA to mediate DSB repair [40], also shows increased transcription in *tertG7* plants.

### Focus on Cell Cycle

Analysis of the regulation of genes related to the control of cell cycle is shown in Table S6. The observed cell cycle slow down in *tertG7* plants (Figure 2) is confirmed by the downregulation of mitotic cyclins (CYCB1;2, CYCB2;1, CYCB2;2, CYCB3;1) and activators of anaphase-promoting complex/cyclosome (APC/C), involved in degradation of mitotic regulators and promoting mitosis and cytokinesis (CDC20;1, CDC20;2) [41]. Cell cycle progression inhibitors are upregulated. This is the case for the WEE1 kinase that is known to be rapidly induced after DNA stress and to interfere directly with cell cycle progression through a mechanism that probably involves inhibitory phosphorylation of the main drivers of the cell cycle, the cyclin-dependent kinases (CDKs) [42]. SMR7 and KRP6 (CDK inhibitors) are also upregulated by the presence of dysfunctional telomeres in *tertG7* plants. We also note that the mitotic cyclin CycB1-1, which has been reported to be upregulated by genotoxic stress [32]–[34], is upregulated in response to telomere damage. Thus, cell-cycle regulators that inhibit CDK activity or cell cycle progression are upregulated, while those promoting mitosis are downregulated.

### Focus on Senescence/PCD

No role of telomeres in plant senescence has been established. No leaf senescence is observed in *tertG7* plants and despite severe morphological abnormalities, late-generation *tert* mutants have an extended lifespan and remained metabolically active [22]. In accordance with these observations, relatively few genes related to senescence show altered expression in *tertG7* plants (Table S7). This result contrasts strikingly with a recent report of the biological consequences of telomere dysfunction in mice. Fourth generation *tert* mice (absence of telomerase+telomere damage) show impaired mitochondrial biogenesis and function, decreased gluconeogenesis, cardiomyopathy, and increased ROS (reactive oxygen species) levels [27]. This mouse study highlights the link between telomere shortening/deprotection and p53-dependent compromised mitochondrial function, driving the premature ageing observed in TERT-deficient mice [27]. The results presented here in this analogous study in plants contrast strikingly with the mouse study, with no significant alteration of mitochondrial related gene expression observed in our *tertG7* plants (Table S8).

Among the cell death related genes, we have however remarked the misregulation of several Lipid Transfer Proteins (LTPs) or LTP-related genes. These proteins are thought to be involved in formation and reinforcement of plant surface layers [43] and in defence against pathogens [44]. Interestingly, it has been shown that a long period of Sucrose starvation induced autophagy in suspension cultures of *Acer* spp. cells [45] and that autophagy was paralleled with a massive breakdown of membrane lipids. In *Euphorbia lagascae* seedlings, localization of LTPs correlates with PCD responses during endosperm degradation [46]. Cell death observed in meristems of *tertG7* mutant plants seems to be related to an autolytic rather than to an apoptotic process. Implication of autolytic process has been reported in radiation-induced cell death in Arabidopsis root meristems [29] and appears to be a general pathway of cell death in plants in response to genomic stress.

## Conclusions

Absence of the telomerase reverse transcriptase (TERT) leads to the progressive erosion of telomeric DNA sequences, which in turn, results in telomere uncapping and increasingly severe genetic instability accompanied by defects in growth and development. This is clearly seen in *tertG7* plants, which show poor growth and seed germination, increased cell death and mitotic slow-down. Given the severe genetic damage visible in these plants, with 37% of mitoses in roots showing at least one visible dicentric chromosome bridge, the “mildness” of the impact of these effects is however striking and these plants remain able to develop. It is only after two or three more generations that *tert* plants become so severely affected that they lose the ability to develop and reproduce (*tert G9-11*) [22], [47].

Telomerase mutant mice show accelerated ageing and severe developmental phenotypes [27], notably including defects in mitochondrial biogenesis and function. Transcriptome analyses ascribe a major role in this for p53-dependent repression of PGC-1alpha and PGC-1ß (peroxisome proliferator-activated receptor gamma, coactivator 1 alpha and beta). As underlined by the authors of the mouse study, this occurs not only in proliferative tissues, where roles of p53 in cell-cycle arrest and apoptosis are well established, but also in more quiescent organs such as heart and brain [27]. In contrast, cell death in Arabidopsis *tert* mutants is mostly restricted to actively dividing meristematic cells, and plants show progressively more severe developmental defects but no accelerated ageing. The “mild” effects on cell division and on gene expression in these plants, notably on mitochondrial genes, concord with these phenotypes and further underscore the contrast with mammals.

Why then are the effects of telomere damage so strikingly different between plants and animals? One possibility comes from the differences in regulation of telomerase expression, limited to dividing cells in plants, but not in mice. We note however, that in the context of our results and those of the mouse study [27], telomerase is not expressed in any cells of the *tert* mutants. Thus in late generation mutants (G4 in mice and G7 in plants), the analysis is of the consequences of the absence of telomerase, not absence of the enzyme itself. Further studies of specific cell types in early generation plants (G2 plants) will be needed to respond to the question of differing effects of the absence of telomerase in dividing versus non-dividing cells of the plant. We suggest that the explanation of these strikingly different effects of telomere damage seems more likely to come from differences between plants and animals in the linkage between the surveillance of genome integrity and the apoptotic response. In mammals, the response to DNA damage is almost exclusively governed by p53, which regulates the critical choice between apoptosis, cell-cycle arrest and cell cycle progression. Notwithstanding the apparent absence of a plant p53 orthologue, the existence of DNA damage-induced, programmed cell death in plants has been well established [19], [29], [48], This response is dependant on the ATM and/or ATR kinases and recent work has shown the SOG1 transcription factor to be required downstream for induction of cell death. Recent reports confirm the importance of ATR in the selectively culling genetically damaged cells due to telomere dysfunction during Arabidopsis development [19], [20]. In contrast to the situation in animals, this programmed cell death response in plants appears to be mostly restricted to dividing meristematic cells. Killing the meristem cells by irradiation however results in the initiation of a new meristem in adjacent tissue and the continuation of growth and development [29], [38], [48]. This developmental plasticity as a response to DNA damage-induced PCD can explain much of the observed radioresistance of plants. Plants also survive major physical traumas, such as loss of limbs, without difficulty and uncontrolled cell division leading to tumours or “galls” is common, but does not have the debilitating and often fatal effects of tumours in animals [49]. It is tempting to speculate that these characteristics have led to selection for a significant damping of the DNA damage-induced cell death response.

## Supporting Information

Figure S1 **Quantitative RT-PCR results are shown for the DDR transcripts PARP1, BRCA1, and RAD51 on 7-days old plantlets.** Expression levels are relative to wild type. n = 3. *p<0.05 relative to wild type (Student’s t-test). Error bars represent SEM.
(PDF)

Table S1 List of 18893 genes and transcription data from two independent RNA-seq experiments.
(XLSX)

Table S2 Lists of genes showing differential expression between *tertG2*, *tertG7* and *WT* plantlets.
(XLSX)

Table S3 Lists of “Telomere damage” and “telomerase” genes.
(XLSX)

Table S4 Lists of genes belonging to the “stress” category. The relative induction is indicated for both RNA-seq experiments.
(XLSX)

Table S5 Lists of genes belonging to the “DNA repair and recombination” category. The relative induction is indicated for both RNA-seq experiments.
(XLSX)

Table S6 Lists of genes belonging to the “cell cycle” category. The relative induction is indicated for both RNA-seq experiments.
(XLSX)

Table S7 Lists of genes belonging to the “PCD/senescence” category. The relative induction is indicated for both RNA-seq experiments.
(XLSX)

Table S8 Lists of genes belonging to the “mitochondrial genes” category. The relative induction is indicated for both RNA-seq experiments.
(XLSX)
