# Supplementary figures and images for: Responses to Telomere Erosion in Plants
Source: PLoS One. 2014 Jan 21;9(1):e86220. doi: 10.1371/journal.pone.0086220 (PMC3897657; doi:10.1371/journal.pone.0086220)

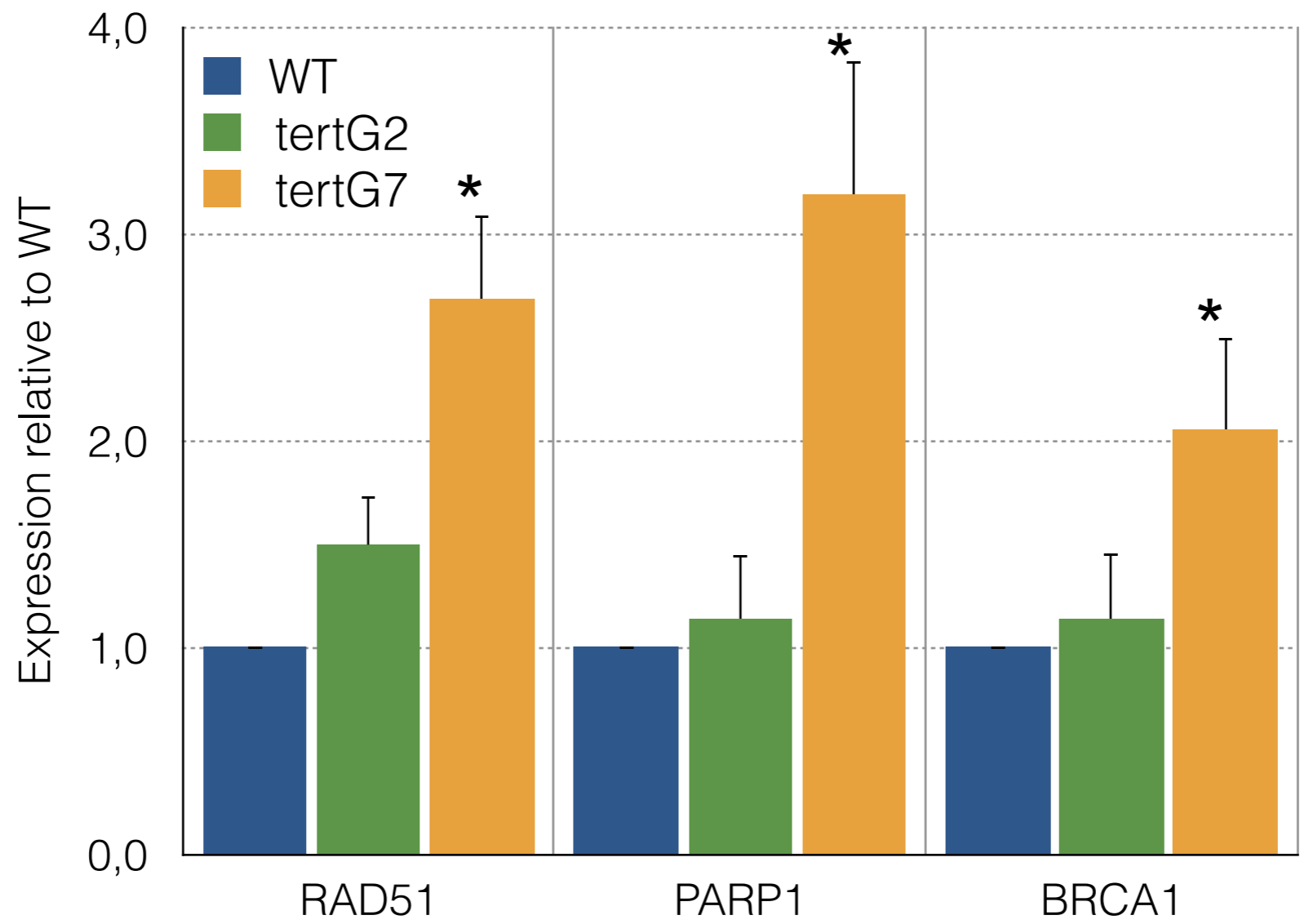

Supplement: Figure S1 — Quantitative RT-PCR results are shown for the DDR transcripts PARP1, BRCA1, and RAD51 on 7-days old plantlets. Expression levels are relative to wild type. n = 3. *p<0.05 relative to wild type (Student’s t-test). Error bars represent SEM. (PDF) [file pone.0086220.s001.pdf]
